# Vaccine Licensure in the Absence of Human Efficacy Data

**DOI:** 10.3390/vaccines10030368

**Published:** 2022-02-26

**Authors:** Courtney L. Finch, Christian Martinez, Elizabeth Leffel, Mario H. Skiadopoulos, Adam Hacker, Betty Mwesigwa, Diadié Maïga, Ian Mugisa, Grant Munkwase, Roxana Rustomjee

**Affiliations:** 1 Sabin Vaccine Institute, Washington, DC 20037, USA; christian.martinez@sabin.org; 2 Leffel Consulting Group, LLC, Eagle Rock, VA 24058, USA; beth@leffelconsultinggroup.com; 3 Microvirion Vaccine Consulting, Rockville, MD 20852, USA; mskiadopoulos@gmail.com; 4 Coalition for Epidemic Preparedness Innovations, Bloomsbury, London NW1 2BE, UK; adam.hacker@cepi.net; 5 Makerere University Walter Reed Project, Kampala 16524, Uganda; bmwesigwa@muwrp.org; 6 Regional Office of Africa, World Health Organization, Brazzaville P.O. Box 06, Congo; maigad@who.int; 7 National Drug Authority, Kampala 23096, Uganda; imugisa@nda.or.ug (I.M.); gmunkwase@nda.or.ug (G.M.)

**Keywords:** filovirus, Ebola, Marburg, vaccine, licensure, regulatory, Animal Rule

## Abstract

Clinical vaccine development and regulatory approval generally occurs in a linear, sequential manner: Phase 1: safety, immunogenicity; Phase 2: immunogenicity, safety, dose ranging, and preliminary efficacy; Phase 3: definitive efficacy, safety, lot consistency; and following regulatory approval, Phase 4: post-marketing safety and effectiveness. For candidate filovirus vaccines, where correlates of protection have not been identified, and phase 2 and 3 efficacy of disease prevention trials untenable, large and/or protracted, each trial may span decades, with full licensure expected only after several decades of development. Given the urgent unmet need for new Marburg virus and Ebola Sudan virus vaccines, the Sabin Vaccine Institute hosted a key stakeholder virtual meeting in May 2021 to explore the possibility of licensure by use of an “animal rule-like” licensure process, based on a risk/benefit assessment specific to regional needs and informed by epidemiology. This may be appropriate for diseases where there are no or limited treatment options, and those prone to sporadic outbreaks with high rates of transmission, morbidity, and mortality. The discussion focused on two contexts: licensure within the Ugandan regulatory environment, a high burden country where Ebola vaccine trials are ongoing, and licensure by the United States FDA—a well-resourced regulatory agency.

## 1. Introduction

Vaccine regulatory processes generally follow similar (often called “traditional”) pathways to licensure; however, not every disease or every candidate vaccine conforms to traditional standards. Traditional approval pathways require preclinical/nonclinical studies to demonstrate the ability of a vaccine to induce the desired immune response and to demonstrate an acceptable safety profile in animals in addition to clinical trials to con-firm safety and efficacy in humans. There are some diseases for which efficacy cannot be fully demonstrated in clinical trials. Either human challenge trials for vaccines are not possible due to a high case fatality rate of the challenge pathogen, or disease outbreaks are too infrequent and too small to gather enough efficacy data to support approval through a field trial. In these scenarios non-traditional pathways, which rely on bridging between human immunogenicity and animal immunogenicity and animal efficacy data, must be employed as the primary avenue for regulatory approval. One example of a non-traditional regulatory pathway fitting this mold has been established in the United States (U.S.) by the Food and Drug Administration (FDA) and is commonly referred to as the Animal Rule [1,2]. Under this pathway, efficacy is established in appropriate animal models and bridging to human data occurs via an immune correlate of protection relevant to the animal model and to humans. We refer to pathways that are reliant on animal efficacy data as “animal rule-like” pathways throughout this manuscript.

Other non-traditional regulatory pathways exist outside the U.S., such as the Ex-traordinary Use New Drugs pathway in Canada and Conditional and Exceptional Cir-cumstances pathways in the European Union (EU) [3,4]. There are core underlying principles across the different regulatory mechanisms no matter how traditional the pathway: safety and efficacy data in animals supported by safety data in humans should suggest that the test article is likely to yield human clinical benefit. Despite the commonalities between traditional and non-traditional pathways, formal animal rule-like regulatory mechanisms are generally uncommon and often do not exist in the countries with endemic diseases against which vaccine efficacy cannot be tested in human trials.

Ebolavirus and Marburgvirus are genera of the Filoviridae family of viruses which cause severe and often fatal disease in humans and nonhuman primates [5,6,7]. They are considered emerging infectious disease viruses and are endemic to countries on the African continent. Ebola virus disease (EVD) affects all age groups with an average case fatality rate of 50%, ranging from 25% to 90% [6]. Marburg virus disease (MVD) also affects all age groups and has a case fatality rate of 24–88% [5]. Human-to-human disease transmission in both cases typically occurs following a zoonotic event (an animal-to-human transmission event) involving human exposure to virus-carrying bats or monkeys and subsequent spread from the initial infected individual to another via direct contact with bodily fluids. Signs of disease are similar and may begin with fever, chills, headache, myalgia, and anorexia and progress to vomiting, diarrhea, hemorrhagic fever, impaired organ function and frequently death [5,6]. Outbreaks of both viruses are typically sporadic and small. For example, there have been roughly 40 outbreaks of ebolaviruses (predominantly in African countries) since the first outbreak was identified in 1976. The total number of cases in a single outbreak has rarely exceeded 400 [6]. While there have been a significant number of cases of Ebola virus Zaire, specifically as a result of the 2014–2016 outbreak in West Africa, that have facilitated direct testing of vaccine efficacy in clinical field trials and ultimately led to vaccine licensure, this outbreak, which resulted in over 20,000 cases, is the exception rather than the rule [6,8,9]. Primary treatment for infection remains supportive care. The relatively infrequent incidence of disease outbreaks for filoviruses, such as Ebola virus, Marburg virus and Sudan virus (a filovirus closely related to Ebola virus that is discussed below along with Ebola virus and Marburg virus), and the severity of associated disease may warrant the use of alternative, animal rule-like regulatory pathways to support vaccine licensure.

The focus of Sabin Vaccine Institute’s (Sabin) current research and development efforts is on vaccines to protect against diseases caused by Marburg virus (MARV) and Sudan virus (SUDV). Sabin has been convening workshops with ‘key opinion leaders’ in the field to discuss regulatory approval pathways for vaccine products against filoviruses and other pathogens for which human efficacy data is unlikely to be obtained. Our first meeting, which inspired this manuscript, was a meeting of over 40 participants from multiple institutions in the U.S. and abroad including institutions with which authors of this manuscript are affiliated as well as others. Among the meeting goals were gaining insights from participants on regulatory alignment and understanding existing regulatory mechanisms to vaccine licensure in the absence of human efficacy data. With these efforts we seek agreement on the need for animal rule-like pathways in countries around the world and alignment on the core requirements of such pathways. Here, in collaboration with participating key opinion leaders, we focus on filovirus vaccines (although this review is relevant to vaccines for other pathogens with similar case fatality and incidence) and offer a review of regulatory pathways in the absence of human efficacy data as well as our thoughts on international regulatory alignment in this area. This review intends to provide a regulatory resource and to serve as a call for international regulatory bodies to make further efforts to facilitate and ensure that all countries have regulatory pathways to enable licensure via animal rule-like pathways. The views presented here are those of the authors and are not intended to represent the views of the institutions and organizations with which the authors are affiliated.

## 2. Available Vaccines for MVD and EVD

In addition to describing licensed vectored filovirus vaccines, below we highlight some unlicensed vectored vaccines that have been tested in clinical trials. The general design approach to each vaccine we discuss is to engineer them to carry the Ebolavirus (EBOV) or MARV surface glycoprotein (GP) gene (encoding the most antigenic protein) in a modified virus vector of different species origin such that the viral vector acts as a delivery system for GP [10,11].

Despite the passing of many decades since the first identified MVD case in 1967 and the first identified EVD case in 1976, relatively few licensed vaccine candidates against filoviruses are available today [5,6]. To date, only two vaccines have obtained broad regulatory approval for use against Ebola virus Zaire. The first of these approved vaccines is Ervebo^®^; approved by the U.S. FDA, European Medicines Agency (EMA) and some African countries [9,12,13] (Table 1). Ervebo is a replication-competent vesicular stomatitis virus-vectored vaccine expressing the surface GP of EBOV Zaire. There is also a two-dose vaccine approved in Europe which was developed by Janssen Pharmaceuticals. The first dose is marketed as Zabdeno^®^ and is a human adenovirus 26 (Ad26)-vectored vaccine against EBOV Zaire [14,15,16,17]. The second dose, marketed as Mvabea^®^, is a booster for Zabdeno and is a replication incompetent Modified Vaccinia Ankara virus (MVA) [14]. Other approved EBOV vaccines have been approved in China and Russia (emergency use only) [18,19,20,21] (Table 1). There are no vaccines approved against MARV. Janssen’s Mvabea comes as close as we currently have to a licensed MARV vaccine, and while it contains an insert for MARV, the Mvabea/Zabdeno prime-boost vaccine is approved for EBOV as noted above.

As for unlicensed EBOV vaccines that have been tested in clinical trials, an EBOV monovalent replication-incompetent chimp adenovirus serotype 3 (ChAd3)-vectored vaccine against EBOV Zaire has been evaluated in humans for safety and immunogenicity as have similar bivalent, EBOV Zaire and SUDV, and monovalent SUDV ChAd3-vectored vaccines. Most related ChAd3-based vaccine trials were initially led by National Institute of Allergy and Infectious Diseases (NIAID) and GlaxoSmithKline and are now led by Sabin [22,23,24,25,26]. These vaccines have shown efficacy in nonhuman primates with a single dose [22]. Other EBOV vaccines tested in clinical trials include two human parainfluenza virus type 3 vaccines expressing the Ebola virus Zaire GP [27,28]. Finally, another chimp adenovirus-vectored vaccine, ChAdOx1 biEBOV, a bivalent vaccine expressing GPs of SUDV and EBOV Zaire, has been tested in humans [29]. Even when candidate vaccines have been shown safe and immunogenic, further clinical development apart from enhancing safety databases stagnates in the absence of an outbreak.

In contrast to EBOV, there are only a few vectored MARV virus vaccine candidates that have entered clinical trials. NIAID sponsored a Phase 1a clinical trial for a mono-valent replication-incompetent ChAd3 MARV vaccine that expresses the surface GP of MARV Angola which is similar to the candidate described above for EBOV and SUDV. This trial was an open-label study to examine safety, tolerability and immunogenicity, enrolling 40 subjects at two different doses and completing in 2019 [30]. No safety concerns were identified after 48 weeks of follow-up. This same candidate has been recently tested in a Phase 1b trial sponsored by Sabin in another 16 individuals, again examining safety and immunogenicity [31]. In nonhuman primates, 100% efficacy has been shown with a single dose [32]. Finally, Janssen, in a Phase 1 trial, tested prime-boost combinations for a multivalent filovirus vaccine in which both prime and boost were multivalent, each carrying a MARV component. The approach was similar to that of Zabdeno/Mvabea; Ad26 and MVA were vectors [33]. No other clinical trials of currently unlicensed vectored MARV vaccines have been completed, although there are several entities working to advance new MARV vaccine candidates to clinical trial.

Although significant progress has been made with regards to EBOV vaccines compared to just 10 years ago, there remains much work to be done to address the unmet need of vaccine protection against other filoviral diseases. History indicates that the relatively low rate of outbreak incidence will continue to make field trials to test efficacy of candidate vaccines challenging. Thus, all regulatory agencies must have animal rule-like regulatory approval processes where animal efficacy data can be used to support registration in the absence of the ability to conduct clinical efficacy trials.

## 3. United States Regulatory Pathway—Food and Drug Administration Animal Rule

The FDA Animal Rule regulations are outlined in section 21 Code of Federal Reg-ulation (CFR) 314.600-650 for drugs and 21 CFR 601.90-95 for biologics [2]. The guidelines, which were finalized in 2002, establish a clear regulatory pathway that was previously unavailable for approval of drugs and biological countermeasures against pathogens for which human challenge trials would be unethical and field trials impractical (often due to reasons of naturally occurring outbreak size and frequency, as previously described). While human efficacy data is not required for approval via this mechanism, a Sponsor must still demonstrate that the product is safe in humans through clinical trials and can be manufactured consistently, according to Current Good Manufacturing Practice (CGMP). All requirements must be proven regardless of the regulatory pathway used for licensure.

There are two specific sets of criteria that a product must meet to obtain approval via the Animal Rule. First, the product must be shown to be efficacious in either improving or preventing a condition considered serious or life-threatening. Second, there cannot be any other regulatory mechanism in the U.S. federal code under which the product could be approved. In other words, the Animal Rule cannot be used by choice and must only be used by necessity due to the absence of other applicable options [2].

The primary difference between this regulatory pathway and other more traditional pathways is that the demonstration of efficacy occurs in well-characterized animal model(s). These nonclinical studies can provide substantial evidence of effectiveness when, and only when, the following conditions apply: first, the product’s pathophysiological mechanism of toxicity or the disease and prevention by the product is well understood; second, the effect (e.g., protection induced by a vaccine) has been demonstrated in greater than one animal model thought to be predictive of human response (a single animal model may be sufficient in cases where the animal model is thoroughly characterized and considered highly predictive of the human response); third, the nonclinical endpoints are demonstrably associated with the desired benefit in humans (ex., a vaccine against a pathogen of high human case fatality that confers protection and yields survival in the animal model or a therapeutic that significantly improves disease signs and reduces recovery time); fourth, the data from the animal and human studies provide clear selection criteria for an effective human dose [2].

In planning a development program utilizing the Animal Rule pathway, the FDA can and should play a critical role at each phase of development. Early planning and communication are key. The FDA strongly encourages developers to communicate with them often, allowing them to provide feedback on data gathered, development plans and study designs to help ensure development stays on track and adheres to acceptance criteria under the Animal Rule [2]. Acknowledging and planning for a longer development program may be necessary because it may take substantial time to characterize the model(s) in pilot and proof-of-concept studies. Perhaps the most critical item (and for which consensus with FDA must be established early) is to define how to establish the relationship between the nonclinical/animal model study endpoint for efficacy and the desired (clinical) benefit in humans (i.e., to determine what the correlate of protection will be) for the purposes of extrapolating human efficacy from animal efficacy data [2]. The correlate of protection may be, for example, vaccine induced antibody titer, and without it the animal efficacy data cannot be bridged to the human data. Overall, the body of animal data required for the Animal Rule is significant.

Following approval of a product via the Animal Rule, there may be post-marketing requirements [2]. Such requirements are an important distinction between the Animal Rule pathway and a traditional route to licensure via human efficacy. For instance, a post-marketing human efficacy trial is a likely requirement in the event that the opportunity arises for such a trial, e.g., in the event of an outbreak or declared emergency [2].

## 4. Other Regulatory Pathways

Regulatory pathways vary from country to country and indication to indication. Aside from the Animal Rule, the FDA, for instance, provides another less traditional vaccine regulatory pathway, Accelerated Approval. Outside of the U.S., Health Canada has an approval process termed Extraordinary Use New Drugs, a pathway similar to the FDA Animal Rule [3,34]. The EMA allows for the possibility of approving drugs without human efficacy data. We review regulatory processes below that are relevant to approval of filoviral countermeasures in the U.S. and abroad choosing specific examples to highlight the complexity of this issue of heterogeneity in regulatory rules across the globe. While differences in regulatory requirements are apparent, commonalities exist: the need to demonstrate clinical safety in Phase 1, expanded safety and immunogenicity in Phase 2+, demonstrated manufacturing under cGMP and scale to be emergency use ready.

The Accelerated Approval process is another pathway available in the U.S., and in some cases, it may be an alternative to the Animal Rule provided appropriate conditions are met [34]. A vaccine would qualify for development by an Accelerated Approval if the disease it is intended to prevent is a serious condition and the vaccine provides a meaningful advantage over existing/accessible medical countermeasures. Under the Accelerated Approval process, effectiveness is demonstrated using a surrogate endpoint with a reasonable likelihood of predicting benefit in the clinic. This surrogate endpoint could be an immune marker in the case of vaccines (e.g., seroprotective titer for chikungunya virus) [35,36]. In the absence of a marker reasonably likely to predict clinical benefit, the Animal Rule must be followed [34].

Europe does not have a regulatory pathway identical to the Animal Rule; however, the EMA has alternative pathways that allow flexibility with regard to the data required to support regulatory approval. For instance, Conditional Marketing Authorization may be obtained if the benefit of the product outweighs the risk, if it is likely that a complete dataset (consistent with requirements for traditional approval) will be obtained post-authorization, if the product addresses an unmet need, and if the product offers immediate patient benefit that outweighs the risk associated with lacking a complete dataset [37,38]. If these criteria are met, Conditional Marketing Authorization is issued for a period of one year and can be renewed annually. The EMA has another pathway called Exceptional Circumstances which allows for approval of a product if a Sponsor is unable to provide data on efficacy due to similar reasons described in the Animal Rule [4]. The EMA approved the Zabdeno/Mvabea vaccines in July 2020 under this regulation [14]. There was no clinical trial conducted during an EVD outbreak; an immunobridging strategy was successfully utilized to predict clinical benefit for the selected vaccine dosing regimen. These pathways may intersect in the pursuit of EMA regulatory approval. For instance, Conditional Marketing Authorization provides an opportunity to gather necessary human data for traditional approval; however, if this is not possible, Exceptional Circumstances may be employed to achieve approval. In an important distinction, where conditional approval is intended to lead to full/standard marketing approval, Exceptional Circumstances approval will not typically lead to full/standard marketing approval [4].

The regulatory pathway to approval of an EVD or MVD vaccine in Uganda, a country that has experienced numerous EVD and MVD outbreaks and one in which Sabin will seek to carry out clinical trials, would occur through the National Drug Authority (NDA)-Uganda. NDA has its own policies for regulatory approval (and adheres to internationally accepted practices and guidelines) by traditional pathways [39]. NDA does not have an animal rule-like policy; however, when NDA does not have a policy, World Health Organization (WHO) policies are followed to fill the gap. NDA also performs an abbreviated assessment and review of a product when the product has WHO pre-qualification (a process described in the paragraph below). While NDA has not previously approved products for market without human efficacy data, NDA does have a provision for approval for emergency use of product under ‘emergency circumstances’, a process that has been successfully employed during the coronavirus disease 2019 (COVID-19) pandemic and is only applicable during an emergency [40]. It is a process analogous to the WHO Emergency Use Listing (EUL, described below). NDA policy serves as an excellent example of the importance of WHO in facilitating regulatory decisions in relevant countries and how uncommon animal rule-like pathways are even in the countries that stand to benefit the most from such policies. As of the date of this publication, these authors are not aware of any African country that has implemented an animal rule-like pathway to regulatory approval.

The WHO has two processes, EUL and the Prequalification Program (PQ), by which to facilitate access to vaccines. These processes can also be instrumental in paving the way for vaccine approval in relevant countries, particularly in cases when there are gaps in a country’s regulatory policies. The EUL is a process which can be used to assess unlicensed vaccines and other products used to prevent or treat diseases that are serious or life threatening with outbreak potential and without efficacious alternatives [41]. It is essentially a risk-based approach and is primarily used during a public health emergency. The PQ is a procedure to review quality, safety and efficacy data of a product for international supply [42]. Suitability of the product for use in relevant countries is also assessed during the PQ process [43]. In this way, WHO ensures that these products meet international standards. In addition to facilitating vaccine access, the WHO has made efforts toward the standardization of clinical trial application and marketing authorization application review processes. The African Vaccine Regulatory Forum (AVAREF) was formed by WHO in 2006 and has been the engine of this standardization in Africa [44]. It serves to connect regulators, ethics committees and vaccine developers from participating African member states (55 total) so that treatment and prevention of major illnesses in Africa can be expedited. The AVAREF has made significant progress towards standardization to improve the efficiency of clinical trial application review through standardization of document templates and guidelines. To date AVAREF has facilitated approval of products for COVID-19, EVD (Ervebo), malaria, tuberculosis and others [44]. AVAREF serves as excellent example of an international organization’s success in bringing countries together to achieve efficient, standardized processes, not entirely unlike what will be necessary to achieve animal rule-like pathway regulatory alignment.

Regulatory authorities such as the U.S. FDA, Health Canada and EMA all have animal rule-like regulatory pathways that enable approval of vaccines in the absence of clinical vaccine efficacy data. Other countries may lack the regulations to enable this or are reliant on a WHO PQ which will likely require more conventional development programs including clinical efficacy data unless there is an ongoing emergency. Implementation and alignment of animal rule-like regulatory pathways are critical and currently largely ignored. Without such pathways, countries with endemic diseases such as MVD and EVD do not have a means of approving vaccines in anticipation of need; consequently, they cannot be appropriately prepared for a future outbreak. Instead, they are left to be reactive, waiting for a large outbreak to generate human efficacy data that may eventually result in vaccine approval.

## 5. Licensure of a Vaccine under the Food and Drug Administration Animal Rule—A Case Study

In 2015, the FDA approved the BioThrax^®^ anthrax vaccine (AVA, anthrax vaccine adsorbed) for a post-exposure prophylaxis (PEP) indication under the Animal Rule [45]. This was the first, and to date the only, vaccine to be approved under this regulatory pathway [46]. AVA was originally licensed in 1970 for pre-exposure prophylaxis (PrEP) to protect against cutaneous anthrax disease caused by infection with *Bacillus anthracis* [47]. Data for the PrEP indication was obtained during a time when there was a sufficient number of cases of cutaneous anthrax in the U.S. to allow for evaluation of efficacy in a clinical trial. Since this vaccine had been licensed for a number of years, there was already a sufficient body of chemistry, manufacturing and controls and safety data available to support the PEP indication. Thus, approval for PEP was mainly focused on bridging animal immunogenicity and efficacy data to human clinical immunogenicity data obtained using a post-exposure prophylaxis immunization schedule, although additional human safety data was also obtained [45,48,49].

As required by the FDA, all four criteria of the Animal Rule (described in Section 3) were satisfied for licensure of AVA for the PEP indication [45,48]. The development of appropriate respiratory anthrax animal models to evaluate vaccines and therapeutics, as well as species-independent immune assays that could be used to bridge animal immunogenicity to human immunogenicity were critical for demonstrating that the use of AVA in a PEP setting would be reasonably likely to confer clinical benefit.

It took a number of years for well-characterized animal models to be developed. Much of this work was conducted or supported by the U.S. Government. These animal models, which included rabbits and non-human primates (NHP), were well-characterized for the pathophysiology of respiratory anthrax disease, disease natural history endpoints, and the immunological markers that could be used as correlates of protection [50,51]. The pathophysiological mechanism of toxicity of the anthrax lethal toxin that is produced during a *B. anthracis* infection and its neutralization by antibodies induced by AVA immunization were also well understood [52]. A correlate of protection, in this case toxin neutralizing antibodies (TNA), had also been previously established and validated. Three species-independent serological assays used to measure TNA responses induced by vaccination of animals and humans were available and were validated for use in measuring antibody levels in rabbits, NHPs and humans, respectively [48,50,51]. This allowed for bridging of the animal immune responses and associated survival probability with the human immune response at a specific timepoint after the first immunization. Thus, the immune response in animals and humans were directly comparable using the same immunological assay [45,48]. The next point for concurrence from the FDA was how to bridge animal immunogenicity and efficacy to the human immune response. This included defining the appropriate time points for measuring the immune responses and defining an acceptable target antibody level associated with a particular probability of survival. While it took considerable time for the sponsor and the FDA to come to a consensus regarding the appropriate endpoints and bridging mechanism, it is thought that these experiences may be applicable to candidate vaccines being developed against other pathogens that cannot be readily tested for efficacy in human clinical trials [45,48].

Lessons learned from licensure of AVA for the PEP indication, which can be applied to future candidate vaccines to be licensed via the Animal Rule are as follows. First, the Animal Rule pathway should not be considered a shortcut to licensure; it took almost 10 years to generate sufficient clinical and nonclinical data to support licensure for the new AVA indication, despite utilizing animal models, bioanalytical assays and a correlate of protection that were well established. Vaccine candidates for other viral or bacterial pathogens may not have these parameters clearly defined, and time must be dedicated to these components of a vaccine development program. Second, species-independent bioanalytical/serological assay development needs to be started early because it can take considerable time to develop, validate and complete FDA review. Serological assays that measure an immune correlate of protection will likely continue to play a role in future vaccine licensure strategies. Finally, communication with the FDA should occur very early and continue frequently throughout the development program. This was critical for the success of the AVA program.

## 6. Discussion

There is great need for all regulatory agencies to have animal rule-like regulatory approval processes where animal efficacy data can be used to support registration in the absence of the ability to conduct clinical efficacy trials, particularly for EVD and MVD vaccines, as well as for others like them. In the absence of such regulatory mechanisms, critical time is wasted working towards regulatory approval in a relevant country by first achieving licensure in a country with a rigorous animal-rule like regulatory pathway in place, such as the U.S., and then applying for EUL or PQ with the WHO. As the example of AVA illustrates, animal-rule like pathways are not shortcuts. They are time-consuming and challenging, even when not navigating regulatory pathways in multiple countries, but do afford the opportunity for registering vaccines in the absence of clinical efficacy data outside of an outbreak. Wasted time will lead to lives tragically and unnecessarily lost.

As we have alluded throughout this manuscript, we envision regulatory alignment to first involve agreement to establish animal rule-like pathways across the globe. Such an effort would ideally be facilitated and coordinated by international organizations such as the WHO. Then, core animal rule-like pathway requirements for licensure would be aligned to ensure consistency of the primary regulatory expectations. To maintain each participating country’s sovereignty and allow for diversity of needs, which will inevitably vary by country, each participating country would be permitted to build from the core requirements. Existing animal rule-like pathways could be used as models. This would allow developers to prepare to meet a single set of requirements rather than individual requirements from every country in which approval will be sought. For instance, developers could choose to follow the guidelines outlined by the country with the most extensive requirements while ensuring that they are also accounting for all relevant countries with less extensive requirements since the core requirements would be the same. Any requirements that are inconsistent between a country with less extensive requirements and a country with the most extensive requirements could be worked into the development plan at the outset since all countries would have a known/existing mechanism for animal rule-like approvals. The alignment we outline would allow for parallel submission of product data to pursue regulatory approval in multiple relevant countries simultaneously.

Implementation and alignment of animal rule-like pathways will inevitably require coordination amongst scientists and regulatory bodies of relevant and ally countries that may already have animal rule-like pathways in place. The success of these efforts will depend on open communication and (to a certain extent) a willingness to mold existing systems into common core regulatory requirements. While an aligned set of regulatory requirements is essential, respect for each participating country’s sovereignty must be maintained, and each participant should be afforded an equal voice. In this way, each participating country would be allowed the independence to make its own decision on final approval for licensure.

We recognize that the alignment we are suggesting is a challenge that will be rife with issues from political to infrastructural to societal and more. Thus, this alignment process must be undertaken with great care and respect for societal and cultural norms and with mindful awareness of the need to communicate effectively with the public who will be most affected by regulatory decisions. Maintaining the status quo is easy, but without any efforts made towards alignment, approval will come too late for far too many of those affected.

Since this manuscript was intended to highlight regulatory mechanisms for pursuing licensure of vaccines without human efficacy data and the importance of adopting animal rule-like regulatory pathways, we elected not to include many other nuances and complexities associated with pursuing vaccine licensure that may have distracted from this purpose. We note them here for awareness. Nuances such as market need, the target population, functional immunity versus identification of a correlate of protection (which will not often represent the totality of the functional immune requirements needed for conferring complete protection), and duration of immunity are all important considerations in pursuing vaccine licensure and tailoring a development plan to a specific vaccine candidate.

## Figures and Tables

**Table 1:** Approved Filovirus Vaccines.

Approved Target Indication	Vaccine Name (Vector)	Sponsor	Approval Status	Vaccine Approval with Human Efficacy Data?
EVD	Ervebo (rVSV) [9,12,13]	Merck	Approved via FDA, EMA, Democratic Republic of the Congo, Burundi, Ghana and Zambia	Yes
EVD	Zabdeno/Mvabea (Ad26/MVA) * [14,15,16,17]	Janssen Pharmaceuticals	Approved via EMA	No
EVD	Ad5-EBOV(Ad5) [18,19,20]	Chinese Academy of Military Medical Sciences’ Bioengineering Institute and Tianjin CanSino Biologics	Approved in China	No
EVD	GamEvac Combi (rVSV/Ad5) [19,21]	Russian Federation	Approved in Russia	No

rVSV: recombinant vesicular stomatitis virus; Ad26: human adenovirus type 26; MVA: Modified Vaccinia virus Ankara; Ad5: human adenovirus type 5; * Mvabea carries nucleoproteins of EBOV, MARV, SUDV and Tai Forest virus (TAFV) [14].

## Data Availability

Not applicable.
